# Silage for upcycling oil from saithe (*Pollachius virens*) viscera – Effect of raw material freshness on the oil quality

**DOI:** 10.1016/j.heliyon.2023.e16972

**Published:** 2023-06-03

**Authors:** Line Skontorp Meidell, Rasa Slizyte, Revilija Mozuraityte, Ana Karina Carvajal, Turid Rustad, Inger Beate Standal, Monika Kopczyk, Eva Falch

**Affiliations:** a Norwegian University of Science and Technology (NTNU), Sverres gate 12, 7012, Trondheim, Norway; b SINTEF Ocean, Brattørkaia 17C, 7010, Trondheim, Norway; c Scanbio Marine Group, Bjugnveien 242, 7160, Bjugn, Norway

**Keywords:** Saithe, Viscera, Residual raw material, Silage, Seafood processing, Fish oil, Antioxidant, Omega-3, NMR

## Abstract

The main objective of this study was to investigate how the freshness of saithe (*Pollachius virens*) viscera affected the quality, composition and yield of oil obtained by silaging. Minced viscera with and without liver were stored separately for up to 3 days at 4 °C before silaging at pH 3.8 for 6 days at 10 °C. An antioxidant mixture was added to evaluate the effect on the lipid oxidation. Oil was extracted thermally from untreated raw material during storage (day 0–3) and after silaging. For oil obtained after silaging of viscera with liver, the oil yields increased significantly when the raw material was stored for more than one day prior to the treatment. Use of fresh raw material (collected at day 0) led to significantly lower oxidation compared to longer raw material storage. After one day of storage, the oxidation was less dependent on the freshness. Silaging with antioxidants resulted in significantly lower formation of oxidation products compared to acid without antioxidants and the most significant differences were observed after one day of storage. Contents of docosahexaenoic acid (DHA) and total omega-3 fatty acids decreased significantly when the raw material was stored for 1–3 days prior to silaging compared to fresh raw material. Results obtained by high resolution nuclear magnetic resonance (NMR) spectroscopy indicated that oxidation of esterified DHA might explain the DHA decrease. The free fatty acid content was highest when fresh raw material was used and was most likely affected by the formation of cholesteryl esters observed in NMR spectra after longer storage. The study shows that although the oil quality is reduced during silaging, processing shortly after catch and use of antioxidants can optimize the quality resulting in less oxidized oil richer in omega-3 fatty acids.

## 1 Introduction

In 2021, almost 70% of the whitefish residual raw material generated on-board the Norwegian deep-sea vessels was discarded at sea, accounting for 77 000 tons of potential valuable raw materials [1]. Heads, viscera (including gonads) and liver from saithe (*Pollachius virens*), Atlantic cod (*Gadus morhua*) and haddock (Melanogrammus aeglefinus*) constitutes the most important fractions as these vessels mainly produce* headed and gutted (HG) whitefish [1]. In these lean species, liver and viscera are the most lipid rich fractions that can be utilized to meet the increasing demand for the health beneficial long chain omega-3 fatty acids for food or feed purposes [2]. However, the degradation processes leading to quality loss starts immediately after catch as these fractions are easily degradable due to high activity by endogenous enzymes, microorganisms and oxidation [3]. These processes lead to increased levels of undesirable oxidation products and free fatty acids (FFA) in the oil after longer storage of the raw material [2,4,5]. Due to this it is crucial to develop optimal preservation strategies on-board the fishing vessel [6]. Silaging is a traditional processing method that can be used to preserve the raw material on-board. It is a simple and well-established method that have been used in fish processing since 1936 [7], and is commonly used to preserve residual raw material in the salmon farming industry and pelagic fisheries in Norway [1]. The process starts with mincing of the raw material into a homogenous mass followed by addition of formic acid and mixing to reduce the pH to below 4 [6]. The reduced pH results in optimal conditions for the endogenous proteases and the silage become liquid [7]. The low pH prevents microbial growth and the silage can be stored for several weeks, usually at temperatures between 2 and 24 °C [7]. To speed up the hydrolysis, the biomass is often heated to temperatures around 40 °C [8]. Later in the process, the silage is usually heated to above 90 °C to separate the oil for applications in fish feed while the protein fractions are usually evaporated to produce fish protein concentrate (FPC). The process results in products rich in bioactive compounds, such as omega-3 fatty acids and peptides, and is especially known as a good source of nutrients for animal feeding [8]. In addition, recent studies have suggested that oil high in omega-3 and peptide-rich ingredients can be obtained from silage for food applications [9,10]. However, further research is needed to evaluate the health risks related to human consumption as traces of chemicals might remain in the produced ingredients.

Despite several advantages with the technology, silage is often regarded as a low value product due to formation of FFA caused by active lipases and degree of oxidation products due to the exposure of oxygen during the process [7,11]. Although it is already well established that the quality of the starting material will influence the quality of the outcoming products during processing [5], no published studies have investigated the effects of the raw material freshness on the quality of oil produced by silaging of whitefish residuals. Variations in size of catches, species, by-catch and weather conditions causes large variations in the availability of raw material on-board and storage of fresh raw material prior to silaging might be necessary [6]. To develop optimal on-board concepts, knowledge about how the quality of the produced silage is affected by the freshness of the starting material is therefore needed.

The main objective of this study was to investigate how the freshness of saithe (*Pollachius virens*) viscera affected the quality, composition and yield of oil obtained by silaging. Furthermore, the study aimed to evaluate the effect of using the whole viscera fraction compared to viscera without liver as out sorting of the liver before silaging and subsequent thermal production of liver oil can be an option on-board. Although the technology for automatic sorting is not currently adapted, it has been suggested as a promising technology for on-board sorting of liver [12]. Thus, an understanding of the effects can provide valuable insight for decision makers in concept evaluation for on-board utilization of the remaining viscera fraction. In addition, use of acid containing an antioxidant mixture consisting of propyl gallate, citric acid, propylene glycol and butylated hydroxyanisole (BHA) was tested to evaluate the effect on the lipid oxidation.

## 2 Materials and methods

### 2.1 Raw material

Fresh, wild caught saithe (*Pollachius virens*) (n = 68) with an average weight and length of 3468 ± 747 g and 67 ± 5 cm was used to collect residual raw materials for the experiment. The fish was caught in the Trondheim fjord (Trondheim, Norway) in February 2021 and was stored on ice after stunning and bleeding performed by local fishers, until delivery to the lab on the same day. The fish was gutted immediately after delivery and within 3 h after catch. The viscera (including stomach, intestines and gonads) were carefully collected into two buckets, one containing Viscera with Liver (VL) and one containing Viscera without liver (V). The raw material in each group was minced separately with a HOBART (AE200) mincer with 10 mm holes to make a homogenous mass and placed in plastic buckets covered with plastic (chlorinated polyethylene) at 4 °C.

### 2.2 Acids and chemicals

SoftAcid Aqua M containing formic acid (85% w/w) and lignosulfonic acid (15% w/w) and SoftAcid Aqua MAB + containing formic acid (85% w/w), lignosulfonic acid (15% w/w) and an antioxidant mixture; propyl gallate + citric acid (0.35% w/w), propylene glycol, butylated hydroxyanisole (BHA, 0.16% w/w) (Borregaard AS, Sarpsborg, Norway) were used in the silage experiment. Chloroform, methanol, hexane, *p*-anisidine, acetic acid, isooctane, pyridine, 2-thiobarbituric acid, sodium sulfite, trichloroacetic acid (TCA) (VWR International AS, Oslo, Norway), sodium thiosulfate, potassium iodide, 1,1,3,3-tetraethoxypropane (TEP), oleic acid technical grade, deuterated chloroform (99.96% D) containing 1% tetramethylsilane (TMS) (Merck, Darmstadt, Germany), copper (II) acetate monohydrate (Thermo Fisher, Kadel, Germany) were used for chemical analyses.

### 2.3 Raw material storage and thermal treatment

The minced raw material was stored in plastic buckets covered with plastic (chlorinated polyethylene) for up to 3 days at 4 °C. Sampling of raw material (100 g) was performed from VL and V at storage day 0, 1, 2 and 3. The raw material was placed in 50 ml centrifuge tubes and treated thermally in a microwave (800 W) to inactivate enzymes and separate the oil. The temperature was measured to ensure a temperature of >90 °C. The temperature was kept at > 90 °C for 10 min by heating the raw material for 2 min every other minute in the microwave. All tubes were centrifuged at 2250×*g* for 10 min and then frozen at – 80 °C. The frozen material was separated into four fractions for later analyses: oil, emulsion, stick water and sludge. Not heated raw material samples (100 g) were also collected each day and frozen at - 80 °C for later analyses.

### 2.4 Silaging of raw materials with different freshness

Minced raw material (1800 g x 2) was collected from VL and V at day 0, 1, 2 and 3 for the silage treatment. Each of the raw materials were divided into two parts in 2 L glass beakers where one part (1800 g) was added formic acid (VL-Silage and V-Silage) and one part was added formic acid containing an antioxidant mixture (VL-Silage-A and V-Silage-A). On each storage day, V-Silage-A and V-Silage were added 34 ml of acid, while VL-Silage-A and VL-Silage were added 28 ml of acid to reach the desired pH of 3.8. The silages were placed in a dark room at 10 °C for 6 days to simulate industrial silaging conditions. The pH was measured and adjusted to pH 3.8 each day. After 6 days each silage was collected in a 1 L Erlenmeyer flask with magnet stirrers closed with aluminum foil. The silages were placed in a heating cabinet at 40 °C for 24 h under continuous stirring (to speed up the liquification). In the next stage, the silages were heated in glass beakers to >90 °C for 10 min as described in 2.3 to inactivate endogenous enzymes and extract the oil. Each silage was collected into a 1 L centrifuge bottle and centrifuged at 2250×*g* for 10 min to separate the mass into four fractions: oil, emulsion, stick water and sludge. The oil was pipetted from the upper layer with a glass pipette and the emulsion layer were carefully collected with a spoon. The stick water was filtered through glass wool and the sludge was collected from the bottom layer. The fractions were weighed and frozen at – 80 °C. All silages were performed in duplicates.

### 2.5 Analyses of chemical composition

Content of dry matter and ash (%) was determined gravimetrically by heating the samples at 105 °C for 24 h and 550 °C for 14 h respectively [13] and was performed in duplicates. The lipid content (%) was determined gravimetrically by extracting the oil according to the method of Bligh and Dyer [14] and was performed in triplicates. Protein content (%) was determined by the Kjeldahl method as described by Abel et al. [15] by multiplying the amount of nitrogen with a fish and meat factor of 6.25 [16]. The analysis was performed in duplicates.

### 2.6 Determination of lipolytic activity

The lipolytic activity was determined in raw material stored for 0–3 days according to the method proposed by Izquierdo and Henderson [17] as described by Bergvik et al. [18]. The samples were suitably diluted in phosphate-citric acid buffer with pH 3.8 or 7 and incubated at 40 °C for 15 min. The results are presented in arbitrary units (U) x 10^3^/g wet weight (WW) as average of three parallels.

### 2.7 Determination of free fatty acids (FFA)

The amount of free fatty acids (FFA) was determined as % oleic acid in the oil according to the method of Bernárdez et al. [19] with modifications. Isooctane was used as solvent instead of cyclo-hexane and the analyses were performed in three to five parallels. Mass balance of FFA was calculated out of volumes (g) of produced oil and lipids in sediments after silaging.

### 2.8 Determination of fatty acid composition

Gas chromatography (GC) was used to determine the fatty acid composition of fatty acid methyl esters as described by Dauksas et al. [20]. The analyses were performed in duplicates and the results are expressed as % of total fatty acids.

### 2.9 Analysis by high resolution nuclear magnetic resonance (NMR) spectroscopy

High resolution nuclear magnetic resonance (NMR) spectroscopy was used to study the lipid classes, content of omega-3 fatty acids and FFA. 120 mg of oil was transferred to 5 mm NMR tubes and dissolved in 0.6 ml deuterated chloroform. ^1^H and ^13^C NMR spectra were recorded on a Bruker Avance 600 MHz spectrometer (Bruker Biospin GmbH, Rheinstetten, Germany) with a cryo-probe operating at a ^1^H frequency of 600.23 MHz and ^13^C frequency of 150.94 MHz at ambient temperature (25 °C). ^1^H Pulse program with the following parameters was used: pulse program zg 30, number of scans 24, time domain 65K, acquisition time 3.0 s, relaxation delay 2 s, dummy scans 2, spectral width 18.03 ppm. Parameters for ^13^C were: pulse program zgig, number of scans 512, time domain 131072, acquisition time 1.83 s, relaxation delay 2 s, spectral width 236 ppm. The ^1^H NMR were run quantitatively (recycling time > 3xT1 for using 30° pulse) for peaks in the glycerol and methyl region, where the longest T1 is reported to be for methyl groups of fatty acids (with T1 of 1.5 s) [21]. ^13^C NMR were not run with full relaxation but for a comparison between samples and for evaluation of changes. The spectra were processed in the software Topspin 4.0.7 and peaks were integrated manually based on chemical shifts found in literature [[21], [22], [23], [24], [25], [26], [27]]. The oil samples were mixed with samples from the same day and treatment prior to analysis and lipid classes are expressed as fatty acid equivalences (% of total fatty acids). Content of omega-3 fatty acids, DHA and FFA are expressed as mole %.

### 2.10 Determination of oxidation products

Primary oxidation products were determined by analysis of the peroxide value (PV) using iodometric titration according to ISO 3969 and the AOCS Official Method Cd 8b-90 b [28]. The analyses were performed in three to four parallels and the results are expressed as meq/kg oil. Secondary oxidation products were determined by the *p*-anisidine value (AV) according to AOCS Cd 18–90 [28] and thiobarbituric acid reactive substances (TBARS) according to Ke and Woyewoda [29]. The analyses of AV were performed in duplicates and TBARS were performed in three to four parallels and expressed as umol/g lipid. The total oxidation value (TOTOX) was calculated based on the results of PV and AV by the following formula: 2 x PV + AV [11].

### 2.11 Statistical analysis

SPSS software (IBM SPSS Statistics 27) was used to perform the statistical analyses. Analyses of variance (ANOVA) and Tukey's post hoc test was applied to determine significant difference between samples, storage times and treatments. The significance level was set to *p* < 0.05. All treatments (thermal treatment, silaging with antioxidants, silaging without antioxidants) were performed in duplicates each storage day. The analyses were performed in the number of parallels described for the specific method x 2 if not other stated. The results are expressed as mean values ± standard deviation (SD). The NMR analyses were performed with 24 scans per sample.

## 3 Results and discussion

### 3.1 Gross composition of raw material

The fraction of viscera with liver (VL) accounted for 17.1 ± 4.4% of the round weight of the fish and the viscera (V) for 13.4 ± 4.3%. Falch et al. [30] reported similar results, where saithe viscera with liver accounted for 20% and the viscera made up 14% of the round weight. The average lipid content in VL was 16.5 ± 3.3% (Table 1), close to Dauksas et al. [20] that found an average lipid content of 20% in cod viscera with liver. V contained 4.1 ± 0.6% lipids, similar to Falch et al. [30,31] that reported a lipid content between 1.9 and 9.1% in saithe viscera without liver depending on the season. The lipid content was significantly higher in VL compared to V. This was expected as the lipids in cod species are mainly stored in the liver [5]. During sampling of minced raw material, it was observed changes in the homogeneity of VL at the different storage days. The homogeneity of the raw material mass increased after longer storage, most likely due to proteolytic activity and liberation of lipids during storage. This can most likely explain the larger standard deviations in the lipid contents in VL the first days, especially at day 0 and 1 (Table 1). However, the lipid and dry matter content seemed to increase after longer storage and was most likely due to evaporation of moisture during the storage period. The protein content varied between 14.4 ± 0.2 and 15.5 ± 1.3% of the wet weight for VL, similar to what was found in cod viscera with liver (14.9 ± 2.3%) by Slizyte et al. [32]. The protein content was higher in V and varied between 16.4 ± 0.4 and 20 ± 0.5%. An increase in protein content and dry matter was observed from day 0 to day 3, most likely affected by evaporation of moisture as observed for VL. The dry matter was significantly higher in VL compared to V and can be explained by a lower moisture content and higher lipid content.

**Table 1:** Gross composition (% of wet weight) of raw material collected at each storage day.

Viscera with liver (VL)	Lipid (%)	Protein (%)	Dry matter (%)	Ash (%)
Day 0	18.6 ± 7.2	14.8 ± 1.0	27.8 ± 1.5	1.6 ± 0.0
Day 1	15.9 ± 6.3	14.4 ± 0.2	29.7 ± 1.7	1.7 ± 0.0
Day 2	16.9 ± 2.0	15.5 ± 1.3	34.3 ± 1.4	1.3 ± 0.1
Day 3	14.7 ± 0.5	14.6 ± 0.3	35.9 ± 4.9	1.3 ± 0.4
Average	16.5 ± 3.3	14.8 ± 0.8	31.9 ± 3.8	1.5 ± 0.2
Viscera (V)				
Day 0	4.1 ± 0.4	16.4 ± 0.4	21.2 ± 0.1	1.6 ± 0.1
Day 1	3.7 ± 0.2	18.7 ± 0.0	23.1 ± 0.3	1.4 ± 0.0
Day 2	4.6 ± 1.4	19.1 ± 0.4	23.9 ± 0.4	1.6 ± 0.1
Day 3	4.1 ± 0.1	20.0 ± 0.5	24.0 ± 0.2	1.5 ± 0.1
Average	4.1 ± 0.6	18.5 ± 1.4	23.1 ± 1.3	1.5 ± 0.1

### 3.2 Fatty acid composition in raw material

The fatty acid composition was analyzed in oil extracted from fresh raw materials of viscera with liver (VL) and viscera (V) (Table 2). Monounsaturated fatty acids (MUFAs) were the most dominating fatty acids in VL, while polyunsaturated fatty acids (PUFAs) dominated in V. Significantly higher contents (% of total fatty acids) of C22:6 *n*-3 (docosahexaenoic acid. DHA), C20:5 *n*-3 (EPA), total omega-3 fatty acids and total PUFAs were found in V compared to VL, while VL contained significantly higher contents of MUFAs. This is most likely due to the higher proportion of phospholipids rich in PUFAs in viscera [30]. Total omega-3 fatty acids made out 30.8 ± 0.1% of the total fatty acids in VL and 36.3 ± 0.1% in V, and was in accordance with what found by Falch et al. [30] in viscera and liver of saithe caught in the Barents Sea. This indicates that both fractions can be utilized as valuable sources of omega-3 fatty acids as the content normally make up 10–35% of the total fatty acids in fish oils [33]. The most dominating fatty acids were C22:6 *n*-3 (docosahexaenoic acid (DHA)), C16:0 (palmitic acid), C18:1 *n*-11 (vaccenic acid) + *n*-9 (oleic acid), C20:5 *n*-3 (EPA), and C20:1 (gondoic acid) (Table 2), which is in agreement with earlier studies [30].

**Table 2:** Fatty acid (FA) composition in oil extracted from the raw material (% of total fatty acids, mean ± SD).

Fatty acid	FA (%) in viscera with liver (VL)	FA (%) in viscera (V)
C14:0	4.4 ± 0.0^A^	2.8 ± 0.0^B^
C14:1	0.3 ± 0.0^A^	0.2 ± 0.0^B^
C15:0	0.5 ± 0.0^A^	0.4 ± 0.0^B^
C16:0	15.9 ± 0.1^A^	17.5 ± 0.0^B^
C16:1	5.9 ± 0.0^A^	4.3 ± 0.0^B^
C17:0	1.1 ± 0.0^A^	1.4 ± 0.1^B^
C17:1	0.3 ± 0.0^A^	0.3 ± 0.0^A^
C18:0	3.4 ± 0.0^A^	4.1 ± 0.0^B^
C18:1*n*-11 + *n*-9	15.1 ± 0.1^A^	15.4 ± 0.0^A^
C18:1*n*-7	3.6 ± 0.0^A^	3.1 ± 0.0^B^
C18:2*n*-6	1.4 ± 0.0^A^	1.2 ± 0.0^B^
C18:3*n*-6	0.2 ± 0.0^A^	0.2 ± 0.0^A^
C18:3*n*-3	1.2 ± 0.0^A^	0.8 ± 0.0^B^
C18:4*n*-3	2.0 ± 0.0^A^	1.2 ± 0.0^B^
C20:0	0.3 ± 0.0^A^	0.3 ± 0.0^A^
C20:1	7.8 ± 0.0^A^	5.1 ± 0.0^B^
C20:2*n*-6	0.3 ± 0.0^A^	0.3 ± 0.0^A^
C20:3*n*-6	0.1 ± 0.0^A^	0.1 ± 0.0^A^
C20:4*n*-6	0.8 ± 0.0^A^	1.8 ± 0.0^B^
C20:3*n*-3	0.2 ± 0.0^A^	0.2 ± 0.0^A^
C20:4*n*-3	0.7 ± 0.0^A^	0.6 ± 0.0^B^
C20:5*n*-3	8.0 ± 0.1^A^	10.8 ± 0.0^B^
C22:0	0.5 ± 0.1^A^	0.6 ± 0.1^A^
C22:1*n*-11	5.3 ± 0.0^A^	3.0 ± 0.1^B^
C22:1*n*-9	0.5 ± 0.0^A^	0.3 ± 0.0^B^
C22:2	0.4 ± 0.0^A^	0.3 ± 0.0^B^
C22:3	0.3 ± 0.0^A^	0.2 ± 0.1^A^
C22:4	0.3 ± 0.0^A^	0.3 ± 0.0^A^
C22:5*n*-3	1.3 ± 0.0^A^	1.2 ± 0.0^B^
C24:0	0.0 ± 0.0^A^	0.0 ± 0.0^A^
C22:6*n*-3	17.3 ± 0.0^A^	21.5 ± 0.1^B^
C24:1*n*-9	0.5 ± 0.0^A^	0.4 ± 0.0^B^
Sum SFA	26.1 ± 0.1^A^	27.2 ± 0.1^B^
Sum MUFA	39.4 ± 0.1^A^	32.2 ± 0.1^B^
Sum PUFA	34.5 ± 0.2^A^	40.6 ± 0.2^B^
Sum Omega-3	30.8 ± 0.1^A^	36.3 ± 0.1^B^

Significant difference (*p* < 0.05) is shown as different letters within each row (^A−B^).

### 3.3 Lipolytic activity in raw material

**Fig. 1:**
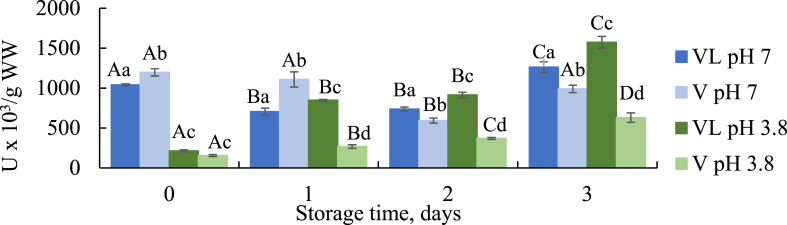
Lipolytic activity as U x10^3^/μmol/g wet weight (WW) in viscera with liver (VL) and viscera (V) during storage. Significant difference (*p* < 0.05) is shown as different letters between days for the same sample (^A−D^) and between samples for the same day (^a-d^).

Lipolytic activity in the raw material of fish leads to hydrolysis of lipids and formation of free fatty acids (FFA) that is an important quality indicator [5,33]. Lipases can be present as endogenous enzymes in the fish but can also be produced by microorganisms present in the raw material [6,34]. To investigate the effect off freshness, lipolytic activity was determined in the raw material of VL and V at storage day 0, 1, 2 and 3. Buffers with pH 3.8 and 7 was chosen to study how the activity was affected by the pH used during silaging compared to natural pH conditions. The temperature at 40 °C was chosen to optimize the hydrolysis conditions in the silage experiment. Significantly higher lipase activity was found in VL compared to V at day 1–3 at pH 3.8 (Fig. 1). This is most likely due to more active lipases in the liver of fish, and is in accordance with a study by Sovik and Rustad [35] that found higher lipolytic activity in cod liver compared to viscera at pH 5. Significantly higher activity at pH 7 compared to pH 3.8 was observed in fresh VL (day 0) and at all storage days for V. This is in agreement with a review by Kurtovic et al. [36] that found the pH optimum for digestive fish lipases to range from 6.5 to 8.5. This indicates that even though high contents of FFA is a common challenge during silaging, the acidic conditions might reduce the lipolytic activity significantly depending on the raw material. A significant increase was observed between each storage day for V and between fresh (day 0) and stored (1–3 days) VL at pH 3.8. The highest increase was seen for VL and might be caused by increased liberation of intracellular acidic lipases located in the liver (with pH optimum close to pH 4) [37]. De Koning [38] reported that the FFA content increased in crude oil during storage due to lipolytic bacteria. Bacterial formation of acidic lipases during storage might also be an explanation. This trend was not observed at pH 7. However, the activity by bacterial lipases is often seen in later stages of storage [34], and 3 days at 4 °C might have been too short to see this effect at natural pH conditions. As lipases are water soluble proteins [36], their breakdown might also occur as the proteolytic activity in viscera are high even at natural pH and low temperatures [3]. Increase in dry matter during storage (Table 1) may also have influenced the results.

### 3.4 Free fatty acids (FFA) in raw material

The amount of free fatty acids (FFA) was analyzed in oil extracted from the raw material during storage and reflects the lipolytic activity [6]. For V, the FFA content was significantly higher at all storage days compared to VL (Table 3). This is most likely due to a lower lipid content in V (3.7–4.6%) where the lipids have been more exposed to lipolysis. Higher amounts of phospholipids in the viscera of saithe compared to the liver [30] might also explain the results as studies have shown faster hydrolysis of these lipids compared to TAG [39]. The FFA content increased and was significantly higher at day 2 and 3 compared to day 0 and 1 for both raw materials. The increase in FFA in the raw material was not reflected in the lipolytic activity measured at pH 7 (3.3) and indicates that analysis of lipase activity might not directly reflect the FFA content. High contents of phospholipids in the viscera [30] and hydrolysis by phospholipase activity, that was not analyzed in the present study, might also affect the results. In addition, the lipolytic activity was measured at 40 °C while analysis of FFA was determined on raw material stored at 4 °C. This has most likely affected the availability of substrate. The enzymatic activity is also highly temperature dependent, and it is unclear if the same trend would occur at 4 °C. However, the measured activity can indicate which raw material that is most exposed to lipolytic breakdown. The time available for the endogenous lipases to hydrolyze the lipids is likely the most important factor for the FFA increase in VL and V, but also the physical conditions in the raw materials and availability of substrate.

**Table 3:** Content of free fatty acids (FFA) given as % oleic acid in lipids extracted from the raw material of viscera with liver (VL) and viscera (V) during storage (mean ± SD).

Storage time, days	FFA (%) in lipids extracted from the raw material of VL	FFA (%) in lipids extracted from the raw material of V
0	1.3 ± 0.2^aA^	3.9 ± 0.2^aB^
1	1.2 ± 0.0^aA^	3.7 ± 0.8^aB^
2	1.7 ± 0.2^bA^	4.8 ± 1.0^bB^
3	2.4 ± 0.2^cA^	5.7 ± 1.0^cB^

Significant difference (*p* < 0.05) is shown as different letters within each column (^a-c^) and each row (^A−B^).

### 3.5 Oil yield after thermal treatment and silaging

**Fig. 2:**
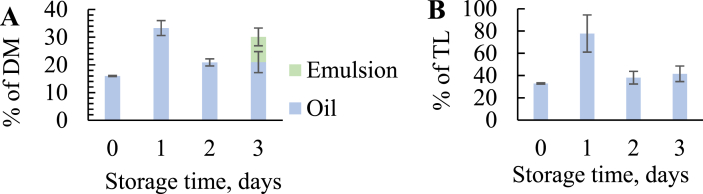
A: Yield of oil obtained after thermal treatment during storage of raw material of viscera with liver (VL) as A: % of dry material (DM) and B: % of total lipids (TL).

The yield of the obtained oil fractions was determined after thermal treatment of stored untreated raw material (VL) and after 6 days of silaging of raw material with different freshness with or without antioxidants (VL-Silage-A + VL-Silage). Due to a low lipid content in viscera without liver (V), ranging between 3.7 ± 0.2 and 4.6 ± 1.5%, no oil was obtained from this raw material during the treatments. For viscera with liver (VL), four fractions were obtained; oil, stick water, sludge and emulsion. Based on % of total dry material (DM) in all fractions, the oil yield was lowest at day 0 (16.0 ± 0.2%) and increased significantly at day 1 (33.3 ± 2.7%), followed by a decrease and lower yields at day 2 (20.9 ± 1.3%) and 3 (21.0 ± 3.8%) (Fig. 2 A). Increase in yield can be seen after storage due to enzymes that liberates lipids from the tissue [5], while a decrease in the yield can occur due to complexes formed between proteins and lipids that end up in the sludge or emulsion [40]. An emulsion layer was seen at day 3, which accounted for 9.1 ± 3.2% of the DM, and might explain some of the decrease in oil. The same trend was observed for total lipids (TL, %) from raw material in the oil fraction, that was lowest at day 0 (32.9 ± 0.6%) and highest at day 1 (77.8 ± 16.7%) (Fig. 2 B).

**Fig. 3:**
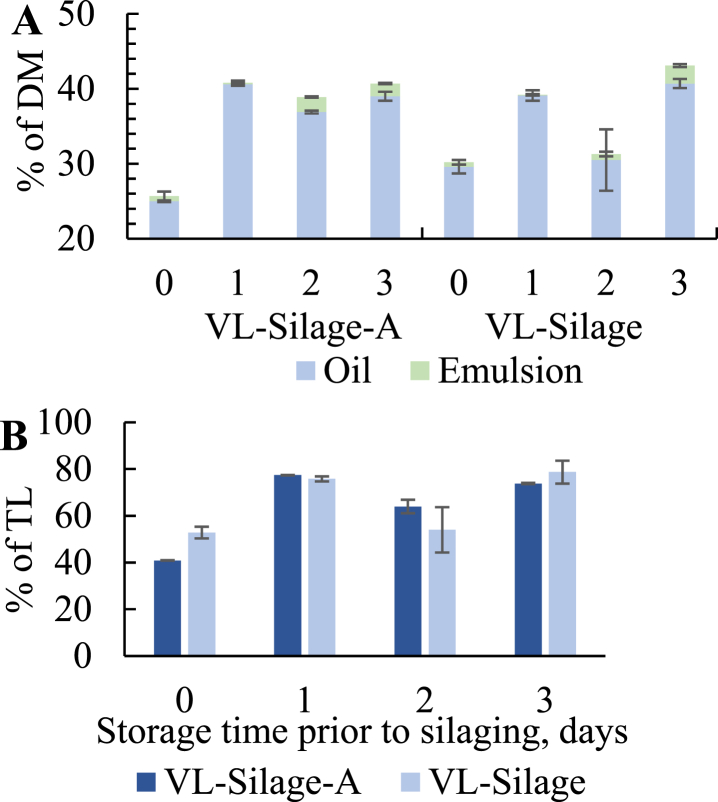
A: Yield of oil and emulsion fractions related to storage time prior to silaging as % of dry material (DM) in silage with antioxidants (VL-Silage-A) and silage without antioxidants (VL-Silage) B: Oil yield as % of total lipids (TL).

The oil yield (% of DM) increased significantly after silaging (Fig. 3 A) compared to the oil obtained from the starting material (Fig. 2 A). This was expected as the proteolytic hydrolysis increases during silaging and due to a longer period (6 days at 10 °C + 24 h at 40 °C) for the enzymes to be active. As expected, addition of antioxidants (VL-Silage-A) did not seem to affect the oil yield. The yield varied between 25.0 *±* 0.1% (day 0) and 39.0 *±* 0.9% (day 3) for VL-Silage-A and between 29.6 *±* 0.9% (day 0) and 40.7 *±* 0.6% (day 3) for VL-Silage. The yield was significantly higher in silage made of stored raw materials (1-3 storage days) for VL-Silage-A and in silage made at day 1 and 3 for VL-Silage compared to fresh materials. For VL-Silage, a decrease was observed at day 2 which also was observed for the raw material (VL) (Fig. 2 A). A thin layer of emulsion was observed for all silages where highest yields of DM were observed for silages made of raw material stored for 2 and 3 days (2.0 *±* 0.1% and 1.7 *±* 0.1% for VL-Silage-A at day 2 and 3, 2.4 *±* 0.2% for VL-Silage at day 3). The obtained lipids in the oil fractions made out 52.8 *±* 2.5% (day 0) to 78.7% (day 3) of the total lipids for VL-Silage and 40.9 *±* 0.1% (day 0) to 77.4 *±* 0.1% (day 1) for VL-Silage-A (Fig. 3 B).

The Bligh and Dyer method [14] was used to determine the total lipids in the sludge fractions of VL-Silage-A and VL-Silage. The lipid content decreased from 25.0 *±* 5.2% at day 0–14.6 *±* 3.2% at day 3 for VL-Silage-A and from 26.5 *±* 2.8% at day 0–16 *±* 1.5% at day 3 for VL-Silage. The lipid content in sludge obtained from silaging of fresh raw materials was close to what has been reported by Slizyte et al. [41] during enzymatic hydrolysis of cod residuals (up to 30%). Significantly lower lipid contents in sludge from silage made of stored raw material (day 3) can be explained by the significantly higher yields of oil obtained from the same silages.

### 3.6 Fatty acid composition in oil and sludge fractions

**Fig. 4:**
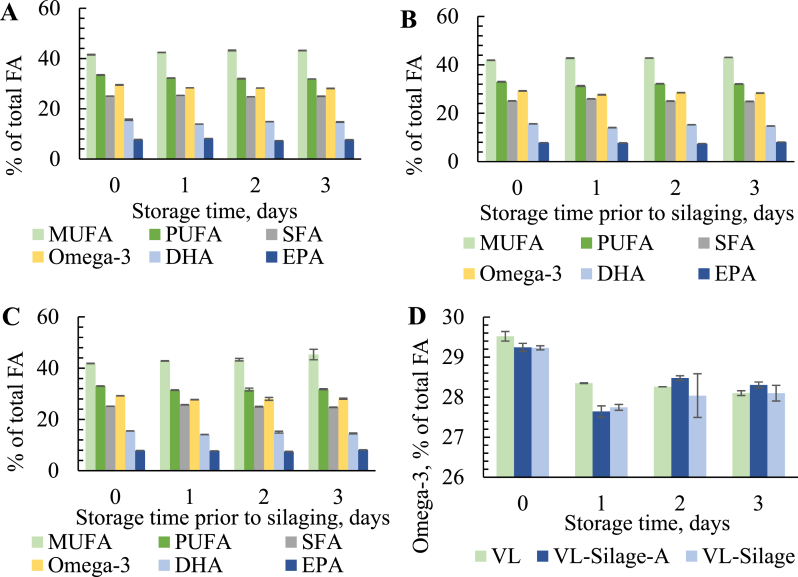
MUFA, PUFA, SFA, Omega-3, DHA and EPA (as % of total fatty acids) in A*:* oil extracted thermally after different storage days (VL)*,* B: oil obtained after silaging with antioxidant (VL-Silage-A) and C: oil obtained after silaging without antioxidant (VL-Silage). D: Content of omega-3 fatty acids in oil obtain from VL, VL-Silage-A and VL-Silage.

The fatty acid composition in oil obtained after thermal treatment of fresh VL (Fig. 4 A) showed that the content of SFA, PUFA and omega-3 fatty acids were lower compared to oil extracted from the raw material with Bligh and Dyer (section 3.2). This difference is most likely due to the extraction methods and that some of the lipids, like DHA located in cell membranes [4], ends in the sludge fraction after thermal treatment and centrifugation. A major part of the phospholipids also tend to end in the sludge [20], where these fatty acids might be located. The most abundant fatty acids in the oil fractions (Fig. 4A–C) were MUFAs, which is in agreement with earlier studies on oil extracted from residuals from Atlantic cod [20]. While marine long chain PUFAs, that was the second most abundant fatty acids, and the omega-3 fatty acids EPA and DHA are well known for their health benefits [42], studies have also shown a possible link between intake of MUFAs from marine oils and prevention of several life-style related diseases [43].

Oil extracted thermally from VL during storage contained significantly higher contents of DHA and total omega-3 fatty acids at day 0 compared to day 1–3 (Fig. 4 D). This was also reflected in oil obtained after silaging where significantly higher contents were found for silage made of fresh raw material compared to 1–3 days of storage prior to the treatment. Due to the unsaturation of PUFAs, these fatty acids are highly prone to oxidation [3], which might explain the observed decrease. Molecular changes during storage might also influence which fraction these fatty acids ends in. It should also be noticed that if oxidation have caused the decrease, this mainly occurred before the silage process and already after 1 day of storage at 4 °C, and that the contents remained stable through the subsequent silage treatment. Sajib and Undeland [44] found a significant decrease in PUFAs and DHA in herring silage after 7 days of storage and discussed lipid oxidation as a possible explanation. A decrease in PUFAs was also found by de Koning [45] in storage of fish oil. Aidos et al. [4] found an opposite trend during storage of herring residuals, were the DHA content increased in oil after storage. This was explained by the liberation of DHA from the cell membranes. Since the decrease was found in the oil fractions in the present study, liberation of DHA into other fractions such as the sludge could also explain the results. The sludge is usually rich in phospholipids with high contents of DHA [20]. Fish lipases and phospholipases have shown to be specific towards hydrolysis of PUFAs and DHA [46], and accumulation of DHA as FFA in the sludge might explain the decrease, especially since significant parts of the FFA tend to accumulate in the sludge [4,47] (section 3.7). To investigate this hypothesis, the fatty acid composition was analyzed in oil extracted from the sludge with the Bligh and Dyer method [14]. The content of omega-3 fatty acids was 27.2 ± 0.8% at day 0 and 23.2 ± 1.4% at day 3 for VL-Silage, and 29.4 ± 0.4% at day 0 and 26.8 ± 0.7% at day 3 for VL-Silage-A. Omega-3 and DHA contents decreased significantly when the raw material was stored for 3 days compared to use of fresh raw material (day 0) for both VL-Silage and VL-Silage-A. Silaging with antioxidants resulted in sludge with significantly higher amounts of omega-3 at both day 0 and day 3. Overall, the results support the possible explanation that parts of these fatty acids might have oxidized during the experiment and that addition of antioxidants had a protective effect against the oxidation.

### 3.7 Free fatty acids (FFA) in oil and sludge fractions

The amount of free fatty acids (FFA) was determined in the oil fractions during storage (VL) and after silaging (VL-Silage-A + VL-Silage) as this is an important quality indicator and is commonly used in pricing of fish oils [11]. For VL, the amount of FFA was significantly higher in oil extracted with Bligh and Dyer (Table 3) compared to thermal treatment (Table 4). Similar results have been reported in earlier studies [20,47], and is most likely due to FFA that ends in the sludge due to the higher polarity of these fatty acids [47]. Like what was found in the raw material, the FFA content in oil extracted thermally increased after longer storage. A significant increase was found between each day of storage. This increase was expected, as liver and viscera in fish contain high amounts of lipolytic enzymes that hydrolyses the fatty acids during storage [35]. A similar increase have been observed during storage of residuals from cod [2] and herring [4]. However, the FFA content was lower in the present study. According to Bimbo, for high quality oil the amount of FFA in crude fish oil should be kept between 1 and 7% [48]. This indicates that the oil obtained (without silaging) was of high quality even after 3 days of storage of raw material at 4 °C (1.7 ± 0.3%). After silaging, the oils contained significantly higher amounts of FFA compared to untreated stored raw material (Table 4). For silage made of fresh raw material (day 0), the FFA content was more than 60 times higher (12.6 ± 0.2%) than in oil obtained from fresh raw material with no acid treatment (0.2 *±* 0.1%). The FFA content varied between 10.0 ± 0.5% and 13.3 ± 0.2% in oils produced after silaging. McBride [49] found similar results (10.8% FFA) in herring silage after 48 h at 37 °C. Since the FFA content in silage has shown to increase with increasing temperatures [7], it can be assumed that significant parts of the FFA was formed during the hydrolysis step (40 °C for 24 h) in the present study, especially as fish lipases have optimum temperatures close to 40 °C [35]. The results also indicated that the raw material freshness seemed to be a less important factor for the FFA content, especially when comparing 1–3 days of storage prior to silaging.

**Table 4:** Free fatty acid (FFA) content given as % oleic acid in oil obtained during storage (VL) and after silaging (VL-Silage + VL-Silage-A) (mean ± SD). Storage time indicates storage day for VL and storage time prior to silaging for VL-Silage and VL-Silage-A.

Storage time, days	FFA (%) VL	FFA (%) VL-Silage	FFA (%) VL-Silage-A
0	0.2 *±* 0.1^aA^	12.6 ± 0.2^aB^	13.3 ± 0.2^aC^
1	0.6 ± 0.2^bA^	11.0 ± 0.3^bB^	10.9 ± 1.0^bB^
2	1.2 ± 0.1^cA^	10.1 ± 0.5^cB^	8.6 ± 0.2^cC^
3	1.7 ± 0.3^dA^	10.0 ± 0.5^cB^	10.3 ± 0.2^bB^

Significant difference (*p* < 0.05) is shown as different letters within each column (^a-d^) and within each row (^A−C^).

High contents of FFA in silage is often addressed as a challenge due to lipolytic hydrolysis [7,11]. However, it has also been stated that the formation is caused by chemical hydrolysis. Reece [47] observed that the FFA increased immediately after addition of formic acid in silage of sprat. The author stated that chemical hydrolysis and the release of FFA from miscible salts under acidic conditions could be the reason. Other studies have also reported that the most significant part of the FFA increase tend to occur the first days [7,47]. McBride et al. [49] found that the FFA content in inactivated (85 °C for 15 m) silage depended on the pH with highest contents at pH 2.0 (10.8%) compared to pH 4.5 (7.9%). An unexpected and significant decrease in FFA was observed in both silages when the raw material was stored before silaging (1–3 days) compared to silaging of fresh raw material (Table 4). Similar results were reported by Sajib et al. [50] who observed a significant decrease in FFA in herring silage during 2 and 4 months of storage and explained this by oxidation of FFA. An unexplained FFA decrease was also observed by El-Ajnaf [51] during silaging of sardine. It is also known that lipoxygenase originating from the fish tissue can oxidize polyunsaturated FFA [52]. In addition, earlier studies have reported that significant parts of the FFA tend to accumulate in the insoluble protein fraction due to the polarity of the FFA [4]. To investigate if an increase could be seen in the sludge, the FFA content was determined in oil extracted from this fraction with the Bligh and Dyer method [14]. The results showed that the content was 19.5 ± 0.9% at day 0 and 19.8 ± 0.6% at day 3 for sludge of VL-Silage-A and 17.3 ± 1.0% at day 0 and 17.6 ± 1.6% at day 3 in sludge of VL-Silage. The contents were significantly higher in the sludge compared to the oil fractions. However, no significant difference was found in relation to the raw material freshness. The FFA contents were similar to results reported by de Koning [45] who found 18% FFA in lipids extracted from the insoluble proteins after acidification. Since the yield of obtained fractions influences the results, the mass balance of FFA was calculated. The results showed that the FFA content in the sludge of VL-Silage-A made out 48.1 ± 1.4% of the total FFA at day 0 and 20.7 ± 1.6% at day 3 and 48.0 ± 5.0% at day 0 and 20.6 ± 1.1% at day 3 for VL-Silage. For sludge obtained by silaging of fresh raw material, the results was close to what reported by Reece [47], that found 60% of the total FFA content in the solid phase after centrifugation of silage. The mass balance results showed that the total FFA content in all fractions decreased with 3.2 ± 0.3% (VL-Silage-A) and 4.6 ± 1.7% (VL-Silage). Possible explanations might be accumulation of FFA in the emulsion or oxidation.

### 3.8 Nuclear magnetic resonance (NMR) analysis of oil and sludge fractions

**Fig. 5:**
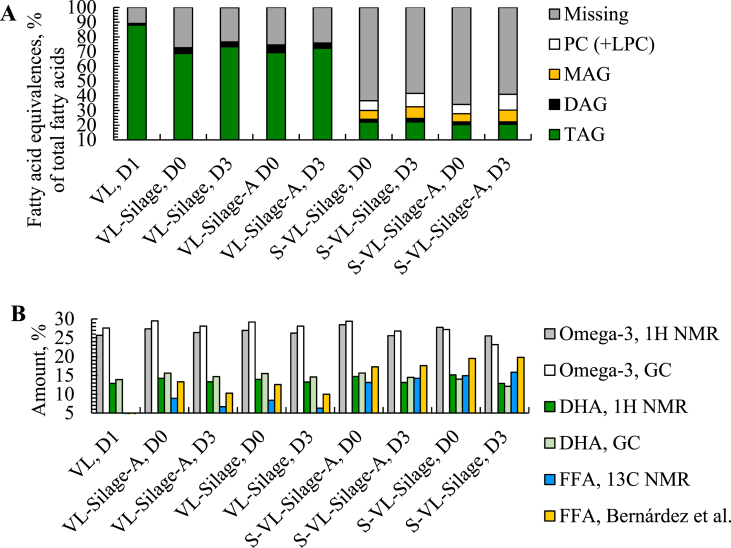
A: Lipid classes as fatty acid equivalences (% of total fatty acids) obtained from ^1^H NMR spectra. B: Omega-3 and DHA content obtained from ^1^H NMR spectra (mole%) and GC (%) out of total FA and FFA contents determined by ^13^C NMR (% of total FA) and by the method of Bernárdez et al. (% oleic acid).

NMR analysis was conducted to study the lipid classes, to confirm the decreases in PUFAs and FFAs in oils obtained after silaging of stored raw material (day 3) compared to fresh (day 0) and to further study possible molecule changes or formation of new molecules. Lipid class determination was based on calculations of fatty acid equivalences out of total fatty acids and regions in ^1^H NMR spectra found in literature [[21], [22], [23]]. Oil obtained thermally prior to silaging (VL day 1) contained 88.0% TAG and 1.1% DAG, close to results reported for oil extracted thermally from cod viscera (93.7% TAG) by Dauksas et al. [20]. Oil obtained from silages made of fresh raw material with and without antioxidants contained 69.3 and 68.7% TAG, 4.6 and 3.9% DAG, and 0.6 and 0.1% MAG. The share of TAG in the produced oil increased to 72.3% (VL-Silage-A) and 73.3% (VL-Silage) when the raw material was stored for 3 days prior to the treatment, while DAG decreased, and no MAG was observed (Fig. 5 A). Increased liberation of TAG from the sludge into the oil during longer storage might explain the increase. This was supported by the lipid classes found in the sludge (S-VL-Silage-A + S-VL-Silage in Fig. 5), where TAG made up a lower proportion of the total classes after longer storage. While spectral regions of phospholipids were below the detection limit for the oils, 6.6–10.7% of the lipid classes in the sludge could be attributed posphatidylcholines (PC) and lysophospatidylcholin (LPC) (hydrolyzed PC) at 3.29 ppm. Significant amounts of phospholipids have also been found in the sludge after enzymatic hydrolysis of cod residuals [20]. Contents of “missing” in Fig. 5A refers to fatty acids not detected as lipid classes. As FFA cannot be quantified in ^1^H NMR spectra, decreases in “missing” fatty acids in oil from silage of stored raw material (3 days) compared to fresh, might reflect the decrease in FFA in the same oils.

Content of total omega-3 fatty acids and DHA was calculated as mole % out of total fatty acids found in the ^1^H NMR spectra according to Igarashi et al. [24]. The results confirmed the GC results (section 3.6) with a decreasing trend in contents of omega-3 fatty acids and DHA in oils when raw material was stored prior to silaging. Good correlations between ^1^H NMR and GC analyses of omega-3 fatty acids have also been reported in earlier studies [25,26]. In addition, normalization and comparison between intensities in ^1^H NMR spectra revealed signals in the region of volatiles such as 2-alkenal and 2-octenal (9.5 ppm) [21,53] in oils obtained by silaging of fresh raw material. No signals were observed in this region in oil obtained after longer storage prior to the treatment. However, intensity in the spectral region of hexanal (9.75 ppm) [53] increased in oils obtained after longer storage compared to fresh raw material. Volatile compounds such as 2-octenal can be degraded to hexanal [54], which indicates that total degradation of such volatiles to hexanal might occurred after longer storage.

Information about the FFA contents were found in the carbonyl region (176–179 ppm) in ^13^C NMR spectra in accordance with earlier studies [21,25]. The FFA changes were evaluated based on the area % of FFA out of signals in the region of all fatty acids (14.06 ppm) [55]. The results confirmed the observed decreases in FFA (section 3.7) in oils obtained from silage of stored raw material compared to fresh (Fig. 5 B). However, a more precise increase in the FFA content in the sludge after longer storage was observed in ^13C^ NMR spectra compared to the method of Bernárdez et al. [19]. The NMR results showed a slightly increase from 14.9 to 15.9% (S-VL-Silage-A) and 13.1–14.3% (S-VL-Silage). The spectrophotometric method of Bernárdez et al. [19] is based on absorption of blue soaps formed with FFA and color complexes in the dark brown oil from the sludge might have affected the detection. Comparison of area % in the carbonyl region of FFA showed that reduction in FFA in the oils was mainly due to reduction in signals of free SFAs/MUFAs and not free DHA, while the increases in the sludge were caused by both free SFAs/MUFAs and DHA. The non-decreasing observations of DHA present as FFA in NMR analysis of the oils might indicate that the decreases in omega-3 fatty acids in oil and sediments obtained after silaging of stored raw material (section 3.6) were caused by oxidation of esterified DHA rather than free DHA. Shen et al. [56] observed that fatty acids esterified to TAG were preferably oxidized over FFA, although it has been stated that FFA oxidize faster than TAG [18,52]. It was observed formation of new molecules in oil obtained after silaging of stored raw material. The signals appeared in the regions of cholesteryl esters at 73.97, 122.65 and 139.67 ppm [27]. Decreases in intensities for cholesterol (121.7 and 140.8 ppm) were also observed. This was also seen in the sludge, but cholesteryl esters were not detected. The results are in accordance with a study by Falch et al. [27] which found esterification of FFA in cholesterol and formation of cholesteryl esters in the same regions as the present study during storage of cod residuals. The action of the enzyme lechitin:cholesterol acyltransferase (LCAT) was discussed as a possible contributor due its activity in forming cholesteryl ester in fish roe. Lovern et al. [39] also observed esterification of cholesterol with FFA to cholesteryl esters during storage of cod.

In general, the results indicates that reduction in omega-3 fatty acids/DHA and FFA in oil obtained after silaging of stored raw material (3 days) compared to fresh (day 0) were caused by different mechanisms. Reduction in DHA in oil and sediments might be explained by autooxidation of esterified DHA to volatiles such as 2-octanal and further degradation to hexanal. This is in accordance with a study by Sajib and Undeland [44] that found significant decreases in DHA while the concentration of hexanal increased significantly after 7 days of storage of herring silage. The decreases in FFA in the oils are most likely caused by formation of cholesteryl esters, some diffusion of FFA into the sludge and possible accumulation of FFA in the emulsion due to its higher polarity.

### 3.9 Oxidative status in oil fractions

**Fig. 6:**
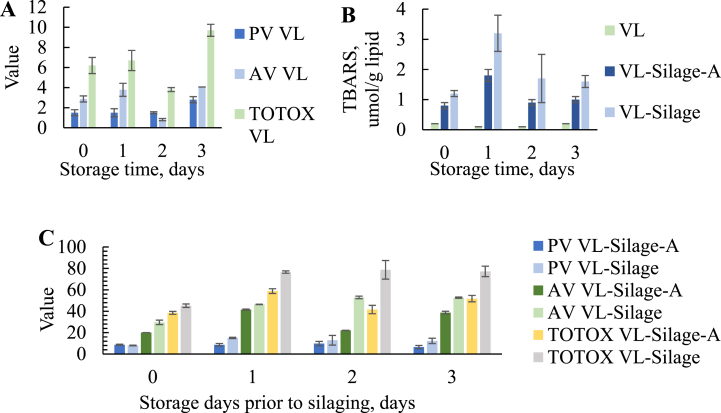
PV (meq/kg oil), AV and TOTOX values in oil obtained thermally from VL during storage (A) and in oil obtained after silaging (C). B: TBARS (umol/g lipid) for VL related to each storage day and VL-Silage-A and VL-Silage related to storage time prior to silaging.

Lipid oxidation was determined in the oil fractions as this is an important factor contributing to quality degradation in fish oils [6]. In oil extracted thermally from VL during storage, the PV was stable the first days and a significant increase was first seen between day 2 (1.5 ± 0.1 meq/kg oil) and 3 (2.8 ± 0.3 meq/kg oil) (Fig. 6 A). The AV increased from day 0 (2.9 ± 0.3) to day 1 (3.8 ± 0.7) and 3 (4.1 ± 0.0). A decrease was observed at day 2 (0.8 ± 0.1) and is most likely caused by the complexity of secondary products formed during oxidation. It is known that oxidation analyses do not always show a linear increase [33], and that secondary oxidation products might react with other compounds [5]. The AV was significantly higher at day 3 compared to all other days. The TOTOX reflected the trend for AV, with an increase from 6.2 ± 0.8 at day 0–9.7 ± 0.6 at day 3. The content of TBARS varied between 0.1 ± 0.0 and 0.2 ± 0.0 μmol/g lipid and no significant changes were observed during the storage (Fig. 6 B). Quality guidelines for refined fish oil used for human consumption provided by European Pharmacopea (Ph. Eur.) recommends a PV ≤ 10 meq/kg oil and AV ≤ 30 [57], while the Global Organization for EPA and DHA Omega-3s (GOED) recommends a PV ≤ 5 meq/kg oil, AV ≤ 20 and TOTOX ≤26 [58]. This indicates that the oils were of high quality and could be used for food applications even after 3 days of storage at 4 °C without the silage treatment.

After silaging, the results of PV and AV were significantly higher than oil extracted from the untreated raw materials (Fig. 6C, Table 5). The PV was significantly lower in VL-Silage-A compared to VL-Silage in silage made at day 1–3 and the AV was significantly lower for all oils obtained from VL-Silage-A compared to VL-Silage, indicating that addition of antioxidants reduced the oxidation. For VL-Silage, a significantly lower PV was observed when the silage was made of fresh raw material compared to storage for 1–2 days prior to the treatment. For VL-Silage-A, the PV increased at day 2 followed by a decrease at day 3. This is most likely due to the breakdown of primary to secondary oxidation products. The AV was significantly lower in silage made of fresh raw material compared to stored raw material (1–3 days) for both VL-Silage-A and VL-Silage. Similar to what was found in VL, a decrease in AV was observed in VL-Silage-A made at storage day 2. This might indicate that the oxidation trend in the raw material can be reflected in silage when antioxidants are added. The TOTOX was significantly higher after silaging compared to untreated raw material. Longer storage time prior to the treatments led to increased TOTOX and significantly higher values were observed when the raw material was stored for 3 days compared to fresh raw material. The results were significantly lower for VL-Silage-A compared to VL-Silage and silaging of fresh raw material led to the lowest TOTOX. The amount of TBARS increased significantly after silaging for both VL-Silage-A and VL-Silage compared to stored raw material (VL) (Fig. 6 B), however TBARS was significantly lower for VL-Silage-A compared to VL-Silage at day 1–3, indicating that the addition of antioxidants limited the oxidation. For both VL-Silage-A and VL-Silage, a significant increase in TBARS was observed in silage made at day 0 (VL-Silage-A; 0.8 ± 0.1 μmol/g, VL-Silage; 1.2 ± 0.1 μmol/g) to day 1 (VL-Silage-A; 1.8 ± 0.2 μmol/g, VL-Silage; 3.2 ± 0.6 μmol/g) followed by a decrease at day 2 (VL-Silage-A; 0.9 ± 0.1 μmol/g, VL-Silage; 1.7 ± 0.8 μmol/g) and 3 (VL-Silage-A; 1.0 ± 0.1 μmol/g, VL-Silage; 1.6 ± 0.2 μmol/g). This might indicate a breakdown of these compounds to tertiary oxidation products. The TBARS values were low compared to an overview provided by Aursand et al. [11], which found TBARS values in fish oils to vary between 0.1 and 3.7 μmol/g lipid. Sajib and Undeland [44] found that determination of TBARS was not a sufficient method to study secondary oxidation products in silage. The authors observed that malondialdehyde (MDA), used in the determination of TBARS, formed acetaldehyde due to its reactivity toward other compounds.

**Table 5:** PV, AV and TOTOX in oil obtained after silaging (VL-Silage + VL-Silage-A) of raw material with different freshness. Storage time indicates days of storage prior to the silage treatment.

	PV (meq/kg lipid)		AV		TOTOX	
Storage time	VL-Silage	VL-Silage-A	VL-Silage	VL-Silage-A	VL-Silage	VL-Silage-A
0	8.0 ± 0.3^aA^	8.7 ± 0.5^aA^	29.6 ± 2.1^aA^	20.1 ± 0.0^aB^	45.6 ± 1.6^aA^	38.5 ± 1.4^aB^
1	15.1 ± 0.6^bA^	8.7 ± 1.2^aB^	46.4 ± 0.2^bA^	41.5 ± 0.5^bB^	76.6 ± 1.1^bA^	58.8 ± 2.2^bB^
2	12.9 ± 4.5^bA^	9.8 ± 2.0^aB^	52.9 ± 1.2^cA^	22.1 ± 0.1^acB^	78.7 ± 8.7^bA^	41.6 ± 3.9^acB^
3	12.3 ± 2.5^abA^	6.5 ± 1.5^bB^	52.7 ± 0.6^cA^	38.7 ± 1.3^bdB^	77.3 ± 4.9^bA^	51.8 ± 3.0^dB^

Significant difference (*p* < 0.05) is shown as different letters within each column (^a-d^) and within the row (^A−B^) of PV, AV and TOTOX.

In general, the oils obtained after silaging were highly oxidized compared to the quality guidelines for PV, AV and TOTOX introduced earlier in this section. The high degree of oxidation was likely caused by a combination of factors, such as the exposure to oxygen and storage time, high contents of PUFAs especially prone to oxidation [3], high contents of FFA that can work as prooxidants [52], oxidation of FFA by lipoxygenase from the fish tissue [52], decrease in antioxidants during silaging and storage [44], and that oxidation tend to increase with higher storage temperatures [33] as the silage was kept at 40 °C the last day of the experiment. According to Sajib et al. [10], oxidation during silaging is dominated by hemoglobin promoted lipid oxidation via heme-mediated cleavage of peroxides. Earlier studies have also reported that acidic conditions increase the activity of prooxidants [5,59]. McClements and Decker [59] found that higher oxidation was observed at pH 3 compared to pH 7 due to iron being more water-soluble at lower pH.

Overall, the results in this study showed that high quality oil could be obtained thermally from saithe viscera with liver before the silage treatment, even after 3 days of storage at 4 °C. Out sorting of liver from the viscera resulted in a low lipid content in the rest fraction and no oil was obtained after thermal treatment or silaging. However, analysis of the fatty acid composition in lipids obtained by solvent extraction showed that this fraction was rich in health beneficial omega-3 fatty acids with a significantly higher omega-3 content out of total fatty acids compared to lipids in viscera with liver. Further research should be conducted to investigate how these lipids can be utilized in an industrial perspective. However, the FFA content was significantly higher in viscera without liver but could be minimized by utilization short time after catch. After silaging of viscera with liver, the oil quality was highly reduced with significantly higher contents of oxidation products and FFA compared to oil obtained prior to the treatment. Raw material freshness and days of storage prior to silaging affected the quality significantly but was less important after more than one day of storage. Use of fresh raw material led to significantly lower contents of oxidation products and significantly higher contents of DHA and total omega-3 fatty acids compared to stored raw material. However, lowest oil yields and highest FFA contents were obtained when fresh raw material was used. NMR analysis revealed increased formation of cholesteryl esters after longer storage which might explain parts of the decrease in FFA after longer storage. This indicates that analysis of FFA might not always be a sufficient method to study quality differences in silage. In addition, NMR analysis showed no decreases in DHA present as FFA after longer storage and indicated that oxidation of esterified DHA might explain the observed DHA decreases. In addition, the results indicated that use of acid containing antioxidants led to significantly lower formation of oxidation products and the highest quality was obtained from silage made of fresh raw material with addition of antioxidants. The study shows that although the oil quality is reduced during silaging, processing shortly after catch and use of antioxidants can optimize the quality resulting in less oxidized oil richer in omega-3 fatty acids. In addition, the results indicates that the raw material freshness seems to be a less important factor if storage for one day or more is necessary.

## Declaration of competing interest

All authors declare that they have no conflicts of interest regarding the submission and publication of the manuscript “Silage for upcycling oil from saithe (*Pollachius virens*) viscera – effect of raw material freshness on the oil quality”.
